# Inflammatory fibroid polyp of the ileum causing recurrent intussusception and chronic ischaemia: a case report

**DOI:** 10.1186/1757-1626-1-244

**Published:** 2008-10-16

**Authors:** Anna M O'Kane, Mark E O'Donnell, Mark McCavert, Kirsty Taylor, Jack Lee, Alan J Wilkinson

**Affiliations:** 1 Department of Surgery, Belfast City Hospital, Lisburn Road, Belfast, BT9 7AB, Northern Ireland, UK; 2 Department of Radiology, Belfast City Hospital, Lisburn Road, Belfast, BT9 7AB, Northern Ireland, UK

## Abstract

**Introduction:**

Inflammatory fibroid polyp is a rare condition of the gastrointestinal tract which can lead to intestinal obstruction.

**Case presentation:**

We present a case of a 65-year-old woman presenting with a 1-month history of intermittent generalised abdominal pain. Computerised tomography imaging demonstrated intussusception of the small bowel. Histology of the resected small bowel revealed an inflammatory fibroid polyp with evidence of chronic ischaemia related to repeated intussusception. This rare complication of inflammatory fibroid polyps is reviewed.

**Conclusion:**

Although computed tomography is useful in confirming an anatomical abnormality, final diagnosis requires histopathological analysis. Operative resection of the affected segment is recommended.

## Introduction

Inflammatory fibroid polyp (IFP) is a rare condition of the gastrointestinal tract. It is a benign solitary or sessile lesion, with an inflammatory basis, originating from the submucosa [1]. IFPs occur most frequently in the stomach and the small bowel and occur rarely in the oesophagus and large bowel. We present a previously unreported complication of a small bowel IFP that caused recurrent intussusception, which compromised vascular supply leading to chronic intestinal ischaemia.

## Case presentation

A 65-year-old lady was admitted with a 1-month history of intermittent generalised cramping abdominal pain with anorexia, weight loss (10 kg), vomiting and loose bowel motions. There were no systemic symptoms of fever, sweating or rigors. Apart from a past history of hypothyroidism and hypertension there was no other significant history and she was a non-smoker. There was no family history of gastrointestinal diseases.

On examination, she was comfortable but dehydrated. She had a heart rate of 107 beats per minute, a blood pressure of 157/84 mmHg and a temperature of 37.2°C. The abdominal examination was unremarkable and bowel sounds were normal. Abdominal X-ray demonstrated multiple air fluid levels but no dilatation of the small bowel or colon. Haematological analyses confirmed a haemoglobin level of 12.4 g/dl, white blood cell count of 8.5 × 10^9^/litre and a C-reactive protein level of 98.5 mg/l. A provisional diagnosis of sub-acute intestinal obstruction was made.

**Figure 1:**
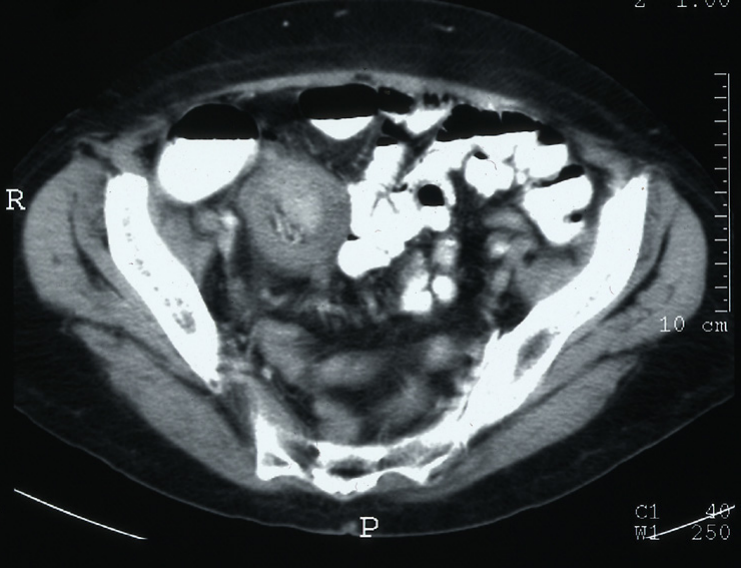
Contrast-enhanced computed tomography scan demonstrating a mass arising from the pelvis with a tubular appearance consistent with small bowel intussusception and mucosal oedema.

**Figure 2:**
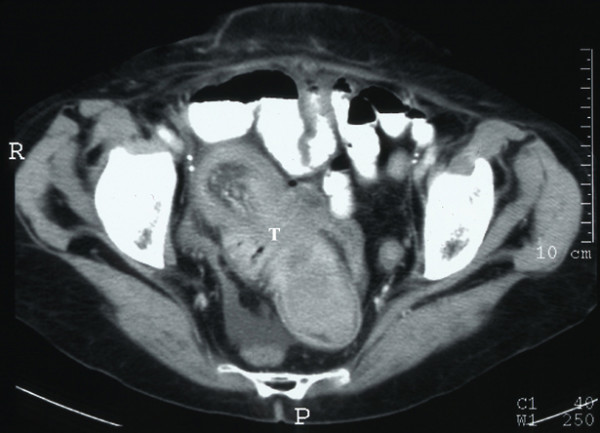
Contrast-enhanced computed tomography scan demonstrating mild dilatation of the ileum proximal to the level of the intussusception with a 'telescoping' appearance of the small bowel intussusception distal to this area (T).

The patient failed to settle with conservative management. Repeat abdominal X-rays confirmed an abnormal air-fluid level mainly in the right upper quadrant. A contrast-enhanced computed tomography (CT) scan demonstrated a mass arising from the pelvis with a tubular appearance consistent with small bowel intussusception and mucosal oedema. There was mild dilatation of the ileum proximal to the level of the intussusception with a 'telescoping' appearance of the small bowel intussusception distal to this area (Figures 1 and 2).

A midline laparotomy confirmed an intussusception approximately 30 cm proximal to the ileocaecal valve with a firm mass at its lead point. The affected bowel segment was irreducible and non-viable. A 30 cm segment of non-viable small bowel was resected and an end-to-end single layer sero-submucosal anastomosis was performed.

Macroscopically, the resected ileal segment contained a 3.5 cm ulcerated pale polypoidal mass 4 cm from the nearest resection margin. The adjacent mucosa demonstrated extensive ulceration, fissuring and incipient necrosis of the wall which was in keeping with chronic ischaemia related to repeated intussusception. Histopathological examination of the polyp revealed an ulcerated polypoidal mass with florid connective tissue proliferation arising from the submucosa and inner muscularis with distortion and reaction of the overlying serosa. Furthermore, there were cytologically bland spindle cells, lymphocytes and eosinophils in the background. There was no evidence of malignancy or necrosis.

The patient's postoperative recovery was uneventful and she was discharged 9 days after surgery. She remains well 20 months later with no further gastrointestinal symptoms.

## Discussion

Only 5% to 16% of all cases of intussusception occur in adults and most surgeons will encounter only a few cases during their careers [2,3]. Less than 1% of all cases of small bowel obstruction in adults are due to intussusception [4]. About 70% to 90% of cases of intussusception requiring surgery have a specific identifiable 'lead point' such as a benign or malignant neoplasm [3,5]. A number of other conditions including Meckel's diverticulum, adhesions, Crohn's disease and coeliac disease have also been identified as aetiological factors [4,6]. However, recent studies using CT and magnetic resonance imaging scans have shown that small bowel intussusception can occur without a demonstrable pathological cause [7]. The majority of intussusceptions are located or begin within the small bowel but colocolonic intussusceptions can also occur [3]. Adults with the condition often present a diagnostic challenge due to atypical symptomatology of varying duration. Although symptomatology depends on the location of the intussusception, the majority of acute patients will present with obstructive bowel sequelae [3]. The chronic indolent course is often attributable to recurrent self-limiting intussusceptions [6].

Plain film radiographs are performed to assess the degree of obstruction and may reveal the presence of free air in keeping with a perforation [2]. Barium contrast studies can show partial or complete intraluminal obstruction. Although ultrasound has been used successfully in the diagnosis of paediatric intussusceptions, its role in adults is less clear as it rarely identifies the underlying cause [2]. CT scanning is the most accurate diagnostic tool as the lead point can usually be defined along with any metastatic disease [7]. There is no consensus on treatment but prompt surgical intervention and resection of diseased bowel is advised for the majority of cases. There is some debate regarding reduction of the intussusception prior to resection but generally this is not advised if malignancy is suspected or when the bowel is inflamed or ischaemic [2]. Lvoff et al. reported that small bowel intussusceptions less than 3.5 cm in length on CT imaging, with no other abnormality, are usually self-limiting [7].

An IFP is a rare, benign, solitary polypoid or sessile lesion which was first described by Vanek in 1949 as a "gastric submucosal granuloma with eosinophilia" and has also been called an inflammatory pseudotumour and eosinophilic granuloma [8]. It arises from the submucosa of the gastrointestinal tract and occurs most frequently in the stomach followed by the small bowel and rarely in the oesophagus and large bowel [1]. Although the exact aetiology is of IFP unknown, it generally evolves from non-neoplastic origins. The maximal incidence of this condition is in the sixth decade of life with an equal sex distribution [9]. Macroscopically, these lesions are usually solitary, measuring between 2 and 5 cm in diameter arising from the submucosa projecting into the bowel lumen. The mucosal surface is often ulcerated and pale. Microscopically, the stroma has a distinctive appearance that is likened to granulation tissue and is diffusely infiltrated with inflammatory cells, mainly eosinophils, although plasma cells are also present [9].

Clinical presentation is determined by the anatomical location. IFPs arising within the stomach produce symptoms of pyloric obstruction or anaemia while those within the small bowel present with obstruction or intussusception [10]. Although radiological investigations are useful in identifying the intussusception, the final diagnosis of small bowel IFP requires pathological confirmation following operative resection. IFPs involving the upper gastrointestinal tract can be resected endoscopically but in cases of small bowel intussusception the treatment of choice is operative resection [1,5,10,11]. There are no reports of death from IFP-associated intussusception but recurrence has been reported [12].

IFP of the ileum causing intussusception has been reported previously [1,5,10,11]. However, none of these reports have highlighted the ischaemic complications that can arise with recurrent intussusception. Intermittent intussusception can compromise mesenteric blood supply to the affected bowel segment leading to bowel infarction, necrosis and perforation [6]. In the case reported here the intermittent abdominal pain appeared to be secondary to recurrent bowel obstruction which compromised vascular supply, leading to ischaemia and subsequent necrosis of the small bowel segment. To the best of the authors' knowledge, this is the first reported case of chronic ileal ischaemia secondary to recurrent intussusception from an IFP.

## Conclusion

Recurrent intussusception secondary to IFPs of the small intestine presents a diagnostic challenge with the potential to cause bowel obstruction, necrosis and subsequent perforation. We have presented a rare complication of IFPs related to repeated ischaemic episodes from an intussusception. Although CT is useful in confirming an anatomical abnormality, final diagnosis requires histopathological analysis. Operative resection of the affected segment is recommended.

## Competing interests

The authors declare that they have no competing interests.

## Consent

Written informed consent was obtained from the patient for publication of this case report and accompanying images. A copy of the written consent is available for review by the Editor-in-Chief of this journal.

## Authors' contributions

AMO was involved in the literature review, manuscript preparation and manuscript editing. MEO was involved in the conception of the report, literature review, and preparation, editing and submission of the manuscript. MM was involved in the literature review, manuscript preparation and manuscript editing. KT was involved in the critical analysis of radiological imaging in the case report and manuscript review. JL was involved in the manuscript editing and manuscript review. AW was involved in manuscript editing and manuscript review. All authors have read and approved the final manuscript.
